# A Myth Still Needs to be Clarified: A Case Report of the Frank's Sign

**DOI:** 10.7759/cureus.2080

**Published:** 2018-01-17

**Authors:** Aung Naing Lin, Kyawzaw Lin, Htoo Kyaw, Joseph Abboud

**Affiliations:** 1 Internal Medicine, The Brooklyn Hospital Center; 2 Medicine, The Brooklyn Hospital Center; 3 Cardiology Department, Cardiology Fellow, Brooklyn Hospital Center; 4 Cardiology Department, Cardiology Attending, Brooklyn Hospital Center

**Keywords:** coronary heart disease, cardiology, frank's sign, bilateral diagonal ear lobe creases

## Abstract

Despite advancements in diagnostic tools, physical signs such as xanthelasmata, arcus corneae, facial wrinkles, and gray hair are useful indicators of underlying diseases. The presence of bilateral diagonal earlobe creases (DELCs), also known as Frank’s sign, correlates with a myriad of cardiovascular diseases such as coronary artery disease, cerebrovascular disease, and peripheral vascular disease. The use of Frank’s sign as a bedside predictor of underlying coronary artery disease is controversial among clinicians. We report a case of a patient with bilateral DELCs found to have significant coronary artery disease during diagnostic coronary angiography for recurrent chest pain.

## Introduction

A diagonal earlobe crease (DELC), also known as Frank’s sign, correlates with a myriad of cardiovascular diseases such as coronary artery disease, cerebrovascular disease, and peripheral vascular disease. The apparent correlation of DELC with cardiovascular conditions was first mentioned in 1973 by Sanders T. Frank [1]. However, its exact etiology remains unknown, and its association with cardiovascular diseases has been questionable.

## Case presentation

**Figure 1:**
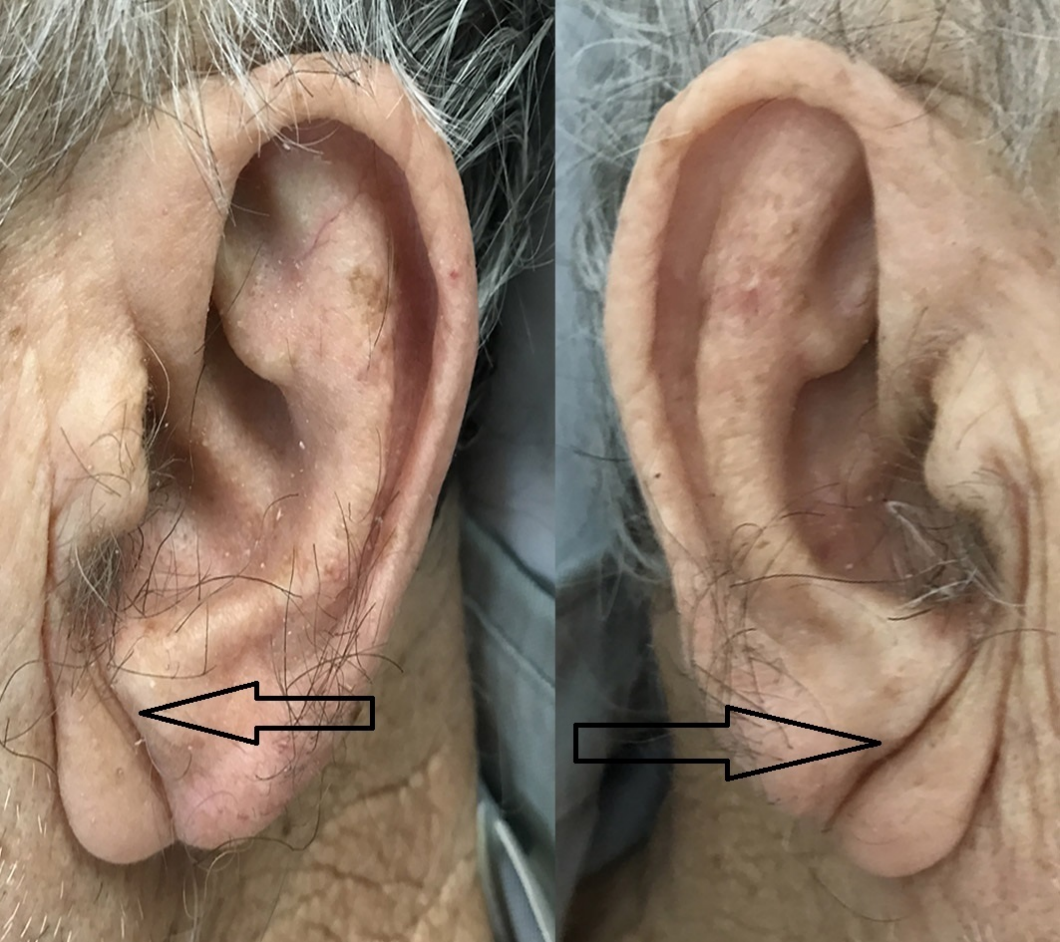
Typical bilateral diagonal earlobe creases (the Frank’s sign) (arrows)

**Figure 2:**
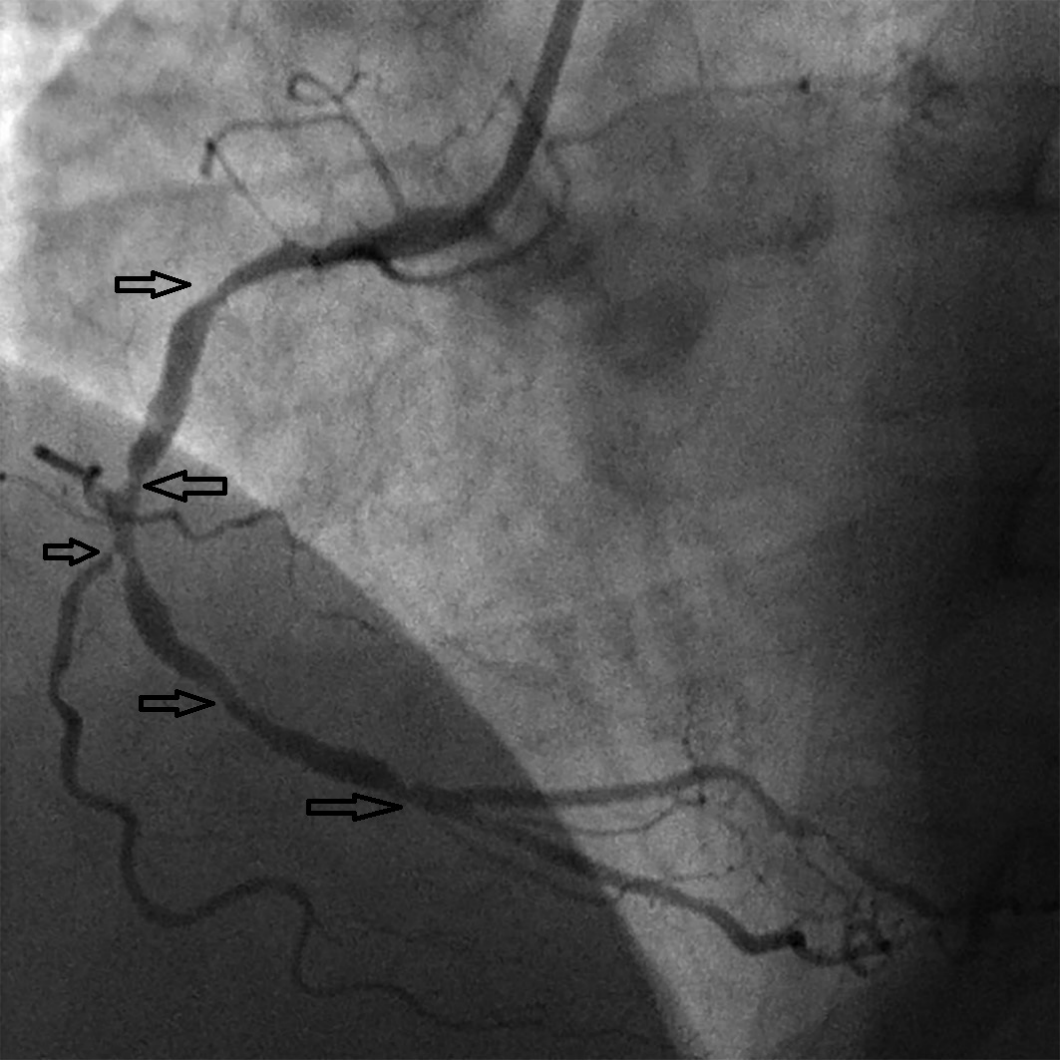
Right coronary artery multilevel narrowing (black arrows) during diagnostic angiography

**Figure 3:**
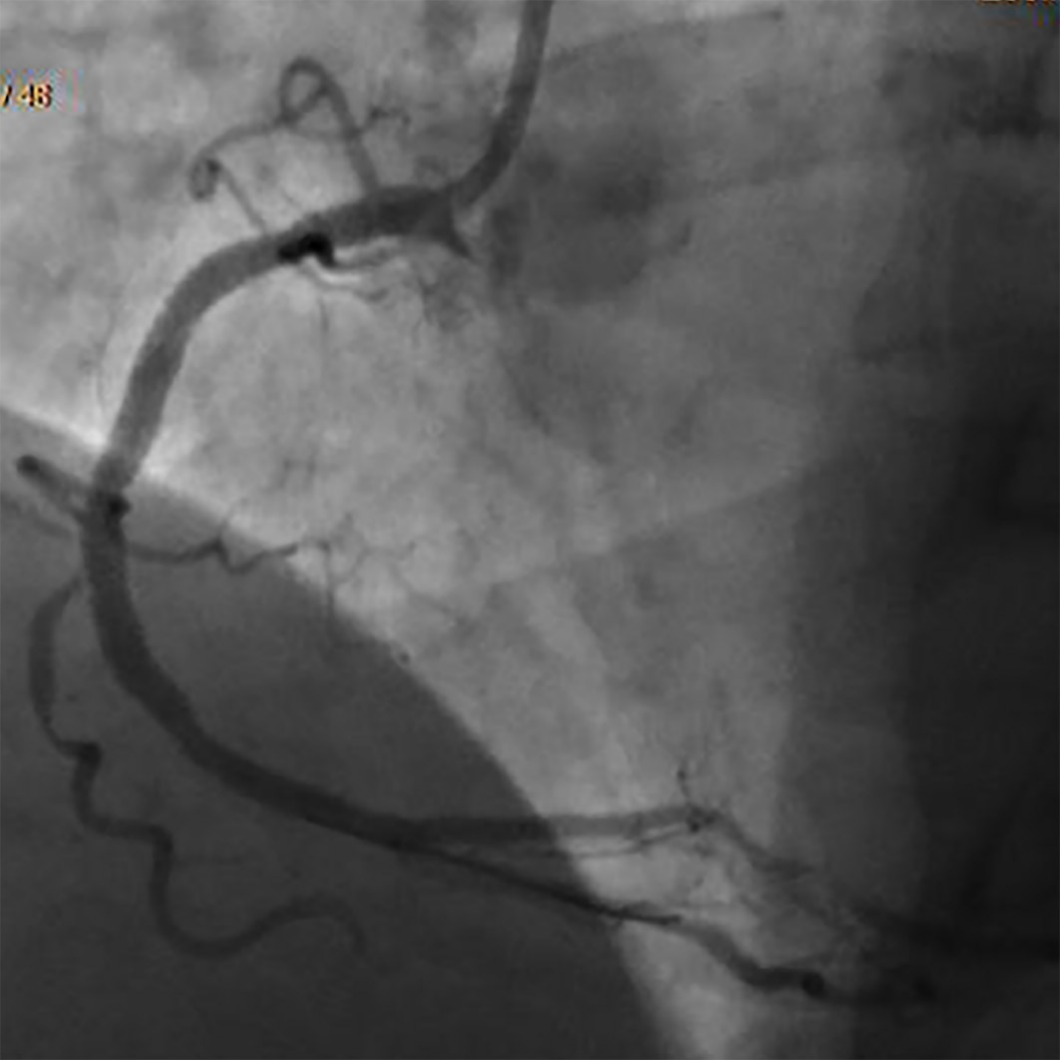
Right coronary artery angiogram after angioplasty and drug-eluting stents placement in the same patient

A 57-year-old man with hypertension and generalized anxiety disorder presented with concerns of a sudden onset of dizziness associated with tightness in his chest, palpitation, and diaphoresis. He stated that his current symptoms were quite different from his usual anxiety attack symptoms. He experienced recurrent pressure-like sensations in his chest. An examination of his vital signs and a physical examination yielded unremarkable findings except for the presence of bilateral DELCs. He stated that he discovered these in his twenties (Figure 1). An electrocardiogram (EKG) showed a normal sinus rhythm with ST depression. His serial cardiac enzyme levels were normal. A subsequent echocardiogram revealed a normal left ventricular ejection fraction of 60% with no wall motion abnormalities. Due to the persistent symptoms and EKG findings, further testing with a coronary angiogram was performed and revealed an 80% occlusion of the right coronary artery (Figure 2). The patient underwent angioplasty, and we inserted drug-eluting stents (Figure 3). 

## Discussion

Diagnostic tools continue to improve, but physical signs such as xanthelasmata, arcus cornealis, facial wrinkles, and gray hair are often indicators of underlying disease. DELCs of varying depths at a 45-degree angle from the tragus to the posterior auricle (i.e., Frank’s sign), is often a dermatological sign indicative of cardiovascular disease. Sanders T. Frank first reported on this correlation between DELC and cardiovascular disease in 1973 [1].

An unbalanced collagen-elastin ratio in the earlobe vasculature and coronary beds is common in patients with DELC. Shortened chromosomal telomeres found in male patients support the relationship between accelerated skin aging and accelerated atherosclerosis (i.e., coronary aging). However, the exact mechanism of Frank's sign in relation to the extent, severity, and prevalence of atherosclerosis is unknown and warrants further exploration [2-8]. Whether DELC is a predictor of underlying coronary artery disease, stroke, and peripheral vascular diseases or merely a trivial sign of aging process is still unclear. The length, depth, bilateralism, and inclination of DELC seem to relate to cardiovascular events [2-9].

While a few previous studies suggest that there is no relationship between DELC and cardiovascular disease, many recent studies report that DELC is an independent predictor of heart diseases regardless of other cardiac risk factors such as hypertension, diabetes, obesity, cholesterol, blood pressure, or smoking. DELC may also correlate with a myriad of other diseases such as cerebrovascular diseases, carotid stenosis, and peripheral vascular diseases in addition to cardiovascular diseases [6-9]. Autopsy findings of patients with DELC further support the association of Frank’s sign to cardiovascular diseases [10].

## Conclusions

Our patient exhibited bilateral DELC and was revealed to have significant coronary artery disease on angiography. While this may not provide a conclusive relationship between DELC and heart disease, our case certainly illustrates that the presence of Frank’s sign may indicate underlying coronary artery disease. Therefore, patients with Frank’s sign should undergo further cardiovascular evaluation.
